# Swine-to-Human Transmission of Influenza A(H3N2) Virus at Agricultural Fairs, Ohio, USA, 2012

**DOI:** 10.3201/eid2009.131082

**Published:** 2014-09

**Authors:** Andrew S. Bowman, Sarah W. Nelson, Shannon L. Page, Jacqueline M. Nolting, Mary L. Killian, Srinand Sreevatsan, Richard D. Slemons

**Affiliations:** The Ohio State University, Columbus, Ohio, USA (A.S. Bowman, S.W. Nelson, J.M. Nolting, R.D. Slemons);; Ohio Department of Health, Columbus (S.L. Page); US Department of Agriculture National Veterinary Services Laboratories, Ames, Iowa, USA (M.L. Killian);; University of Minnesota, Saint Paul, Minnesota, USA (S. Sreevatsan)

**Keywords:** Influenza A virus, swine, public health, livestock, exhibits, zoonotic infections, fairs, zoonoses, H3N2v, viruses, Ohio

## Abstract

Agricultural fairs provide an opportunity for bidirectional transmission of influenza A viruses. We sought to determine influenza A virus activity among swine at fairs in the United States. As part of an ongoing active influenza A virus surveillance project, nasal swab samples were collected from exhibition swine at 40 selected Ohio agricultural fairs during 2012. Influenza A(H3N2) virus was isolated from swine at 10 of the fairs. According to a concurrent public health investigation, 7 of the 10 fairs were epidemiologically linked to confirmed human infections with influenza A(H3N2) variant virus. Comparison of genome sequences of the subtype H3N2 isolates recovered from humans and swine from each fair revealed nucleotide identities of >99.7%, confirming zoonotic transmission between swine and humans. All influenza A(H3N2) viruses isolated in this study, regardless of host species or fair, were >99.5% identical, indicating that 1 virus strain was widely circulating among exhibition swine in Ohio during 2012.

## Abstract 2

Local health care providers should be alerted to the possibility of human infection with variant influenza A viruses, especially during fairs.

In the United States during 2012, approximately 300 cases of human infection with influenza A(H3N2) variant (H3N2v) virus were reported; they resulted in 16 hospitalizations and 1 death (*1*). The variant designation (swine-origin influenza A virus infecting humans) of these cases must be acknowledged because interspecies transmission of influenza A virus plays a substantial role in the evolution of influenza A viruses that infect swine and humans (*2*,*3*). Genomic reassortment resulting in novel influenza A viruses can occur in swine because they are susceptible hosts for avian and human strains as well as strains endemic among swine (*4*,*5*). Thus, swine play a critical role in the ecology and emergence of influenza A viruses that affect human health, as illustrated by the emergence of the 2009 pandemic influenza virus (influenza A[H1N1]pdm09 virus), a reassortant virus with origins that have been traced to influenza A viruses circulating among swine in North America and Eurasia (*6*–*8*).

Bidirectional transmission of influenza A viruses between swine and humans is facilitated by unique swine–human interfaces such as agricultural fairs, where swine from multiple sources commingle with human exhibitors and visitors (*9*). In 2007, novel influenza A viruses, including those of nonhuman origin, became part of the National Notifiable Diseases Surveillance System, and before 2012, outbreaks of variant influenza A virus were reported only occasionally in the medical literature (*10*–*13*); these cases were frequently linked to human exposure to swine at agricultural fairs (*14*–*16*). Epidemiologic investigations by public health officials into human cases of influenza virus subtype H3N2v infection that occurred during 2012 concluded that swine exposure at agricultural fairs was the primary source of the viruses (*17*–*19*).

During the investigation of the 2012 outbreak of influenza A(H3N2v) virus infection, public health officials in Ohio documented 107 confirmed human cases, second only to the number of cases reported from Indiana. In late July 2012, interspecies transmission (from swine to humans) of swine-origin influenza A(H3N2) viruses containing the matrix gene from the influenza A(H1N1) pdm09 virus (H3N2pM virus) was initially confirmed at 1 Ohio agricultural fair (*20*). Retrospective epidemiologic investigations of all subtype H3N2v virus cases determined that human-to-human transmission of subtype H3N2v virus was limited and that most human patients had been directly or indirectly exposed to swine at a total of 14 agricultural fairs across the state (*19*). We investigated influenza A virus activity among swine (the swine side of the swine–human interface) and describe the results of active influenza A virus surveillance among swine at Ohio agricultural fairs during the entire 2012 fair season. When combined with the results of the public health epidemiologic investigation, our data provide molecular corroboration that swine-to-human transmission of influenza A(H3N2v) virus occurred at multiple agricultural fairs.

## Materials and Methods

During 2012, a total of 40 agricultural fairs geographically distributed across Ohio were enrolled in the study. They represented a base of 22 fairs sampled in 2011 that were selected with predetermined criteria and supplemented with 18 fairs randomly sampled in 2012 (*9*). At the end of each fair, study team members visually examined the swine for signs of respiratory disease and collected nasal swab samples from at least 20 swine that were selected without regard to visually determined health status (healthy or ill). Nasal swab samples were placed in individual vials containing viral transport medium and frozen at –70°C until the time of testing. The Ohio State University Institutional Animal Care and Use Committee approved the use of animals in this study under protocol no. 2009A0134.

Detection and characterization of influenza A virus from nasal swab samples were performed as previously described (*21*,*22*). Briefly, samples were screened by real-time reverse transcription PCR (rRT-PCR) for influenza A virus (VetMAX-Gold SIV Detection Kit; Applied Biosystems, Austin, TX, USA). If >1 sample from a fair was positive for influenza A virus, then viral transport medium for all nasal swab samples from that fair was inoculated individually into serum-free medium-adapted MDCK cells for virus isolation (*21*). Cells were observed daily for 72 hours, at which time cell culture supernatant was tested for hemagglutination activity. Hemagglutinin and neuraminidase subtype determination, along with matrix gene lineage characterization, were performed on virus isolates with rRT-PCR by using a commercially available swine influenza viral subtyping kit (Applied Biosystems).

Previously described procedures were used to generate full-length nucleotide sequences for 2 representative swine-origin H3N2pM virus isolates from each fair (*20*). Two isolates from Fair D had previously been sequenced and reported (*20*). All gene segments of the 18 remaining swine-origin isolates underwent amplification by PCR, followed by purification of the cDNA and preparation of cDNA libraries. Quantitated libraries were diluted and pooled for library amplification. After enrichment, DNA was sequenced and the sequences were assembled by using standard procedures. Sequences from the swine-origin influenza A virus isolates reported here have been deposited in GenBank (Technical Appendix).

Of the 14 Ohio fairs that Jhung et al. epidemiologically linked to cases of subtype H3N2v virus infection in 2012 (*19*), 7 (Fairs D–J) had participated in our active influenza A virus surveillance among exhibition swine during the same year (Table 1). As part of the public health investigation into the outbreak of subtype H3N2v virus infections, specimens collected from humans with suspected cases of subtype H3N2v virus infection were submitted to the Ohio Department of Health laboratory for influenza testing. Representative samples with preliminary test results consistent with H3N2v virus infection were forwarded to the Centers for Disease Control and Prevention for confirmatory testing and sequencing. Sequences of the human-origin subtype H3N2v virus isolates were retrieved from the EpiFlu database (http://www.gisaid.org). The human-origin influenza A virus isolates with full-length complete-genome sequences were then categorized by the fair with which the case had been associated during the epidemiologic investigation. Isolates from cases that were associated with >1 fair were removed from the study. This process identified at least 1 human-origin subtype H3N2v virus isolate per fair; if multiple isolates were identified, 1 isolate per fair was randomly selected for further analysis.

**Table 1:** Influenza A virus–specific results, 40 agricultural fairs, Ohio, USA, 2012*

Fair	Week of fair season	Length of swine exhibition, d	ILI among swine reported	No. swine sampled	Positive by rRT-PCR, no. (%)	Positive by VI, no. (%)	Virus subtypes recovered	Associated with H3N2v in humans
A	1	5	Yes	20	14 (70)	5 (25)	H1N1pM and H3N2pM	No
B	4	5	No	20	13 (65)	9 (45)	H3N2pM	No
C	6	4	No	20	6 (30)	6 (30)	H3N2pM	No
D	7	7	No	34	31 (91)	29 (85)	H3N2pM	Yes
E	7	5	Yes	40	39 (98)	28 (70)	H3N2pM	Yes
F	8	5	No	20	20 (100)	18 (90)	H3N2pM	Yes
G	8	7	Yes	20	14 (70)	16 (80)	H3N2pM	Yes
H	8	6	No	20	20 (100)	18 (90)	H3N2pM	Yes
I	9	4	No	20	17 (85)	15 (75)	H3N2pM	Yes
J	10	4	Yes	20	20 (100)	17 (85)	H3N2pM	Yes
30 other fairs	NA		NA	600	29 (5)	0	NA	No
Totals	NA		NA	834	223 (27)	161 (19)	NA	NA

*Real-time reverse transcription PCR (rRT-PCR) and virus isolation (VI) assays performed on nasal swab samples collected from swine at the end of the fairs. Additional details (week of the fair season, clinical signs of influenza-like illness [ILI], and influenza A virus subtypes) are shown for 10 agricultural fairs from which influenza A virus was isolated from >1 pig. Pigs at the other 30 fairs were negative for influenza A virus by VI. NA, not applicable.

Comparative genome analysis was conducted by using the full-length sequences of 1 human-origin and 2 swine-origin subtype H3N2 virus isolates per fair (total of 27 influenza A virus isolates) (Technical Appendix). These sequences were combined with all unique complete influenza A virus gene segment sequences from swine from North America that were available in the Influenza Resource Database (*23*). The nucleotide sequences of each segment were aligned individually by using Geneious version 6.0.5 (Biomatters Limited, Auckland, New Zealand); phylogenetic trees were generated by using maximum-likelihood methods (*24*), and the resulting trees were edited with MEGA version 5.2.2 (*25*). Full-length sequences for each isolate were concatenated to form a 13,133-nt sequence for each isolate; these sequences were then used to examine the genetic distance (number of nucleotide differences) between each isolate. The estimates of genetic distance were calculated by using MEGA version 5.2.2.

## Results

**Figure 1:**
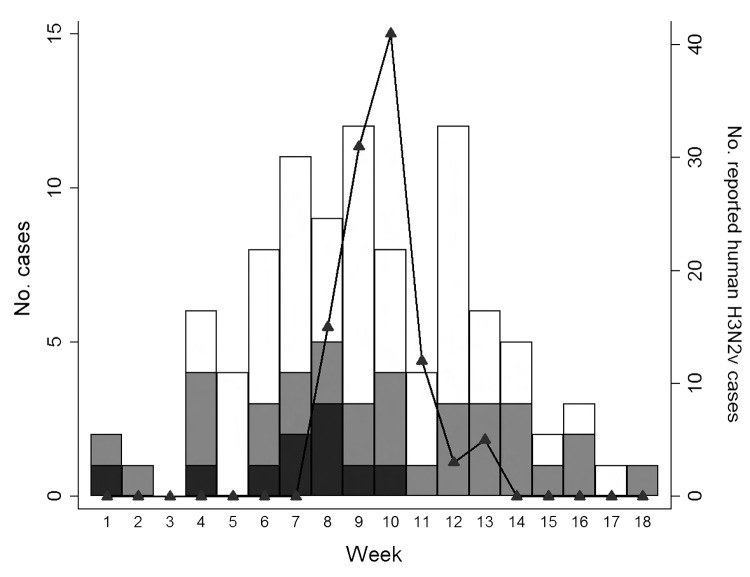
Distribution of agricultural fairs and human infection with influenza A variant virus (H3N2v), by week of the Ohio fair season, June–October 2012. Black bar sections, fairs with swine positive for influenza A virus; gray bar sections, fairs with no swine positive for influenza A virus; white bar sections, fairs not enrolled in this study. Black triangles, reported human cases of H3N2v virus infection.

Influenza A virus was isolated from >1 pig at 10 (25%) of the 40 agricultural fairs. Influenza A(H3N2pM) virus was recovered from swine at all 7 fairs that had been epidemiologically linked to cases of human infection with subtype H3N2v virus. The 30 fairs at which influenza A virus was not isolated from swine were not linked to any cases of human infection with subtype H3N2v virus. The 7 fairs in this study that were associated with cases of influenza A(H3N2v) virus infection in humans occurred during 4 consecutive weeks (weeks 7–10) of Ohio’s 18-week agricultural fair season (June–October) (Figure 1). The 3 fairs at which swine were shedding influenza A(H3N2pM) virus not associated with any cases of human influenza A(H3N2v) infection (Fairs A, B, and C) were held during weeks 1, 4, and 6, respectively. As shown in Table 1, a total of 834 swine were sampled, and influenza A virus was recovered from 161 (19.3%). Although influenza A(H1N1) and A(H3N2) viruses were recovered from exhibition swine during 2012, most (158 [98.1%] of 161) of the isolates were subtype H3N2pM viruses. All isolates, including the 3 subtype H1N1 virus isolates, contained a matrix gene derived from the A(H1N1)pdm09 virus.

The overall nucleotide identity of all 27 isolates included in the investigation was >99.50%. The mean number of differences between each human-origin influenza A(H3N2v) isolate and its 2 matched swine-origin isolates was 9.7 nt (95% CI 2.7–16.7). Conversely, the mean number of differences between each human-origin influenza A(H3N2v) isolate and the 18 swine-origin influenza A(H3N2pM) isolates to which it was not linked was 36.9 nt (95% CI 28.94–44.80). At 5 of the 7 fairs from which human cases of subtype H3N2v infection were reported, the nucleotides of the matched subtype H3N2 isolates from humans and swine were >99.90% identical. Nucleotide identities of 99.74% and 99.87% were detected between the matched subtype H3N2 isolates from humans and swine at Fairs E and F, respectively. Pairwise comparisons of nucleotide differences within and between fairs are shown in Table 2.

**Table 2:** Genetic distances between influenza A(H3N2) viruses isolated from swine and humans at 10 agricultural fairs, Ohio, USA, 2012*

Fair	Fair A	Fair B	Fair C	Fair D	Fair E	Fair F	Fair G	Fair H	Fair I	Fair J
Fair A	4.00	3.38	3.51	6.74	5.59	5.30	7.76	5.00	7.10	7.42
Fair B	19.00	2.00	1.68	5.75	5.15	4.68	7.09	4.12	5.90	7.02
Fair C	17.50	6.00	1.00	6.03	4.48	4.09	6.81	3.84	5.50	6.63
Fair D	48.17	42.17	44.67	2.67	3.53	5.22	5.17	4.83	5.11	5.24
Fair E	48.17	42.17	44.67	31.33	35.33	3.13	3.11	3.47	3.17	3.43
Fair F	34.33	34.17	34.50	32.67	31.67	10.67	4.96	4.56	3.90	5.01
Fair G	53.83	47.83	50.33	33.00	25.00	38.33	2.67	5.43	5.22	1.46
Fair H	31.50	15.50	14.00	32.67	32.67	38.00	38.33	0.00	4.33	5.44
Fair I	47.83	41.83	44.33	27.00	26.00	14.33	32.67	32.33	2.67	5.35
Fair J	55.50	49.50	52.00	34.67	26.67	40.00	4.33	40.00	34.33	6.00

*Full-length concatenated genomes (13,133 nt positions) were used for comparisons. Mean numbers of base differences between isolates within each fair are shown on the highlighted diagonal; mean numbers of nucleotide differences between groups of isolates recovered from each fair are shown in the area below the diagonal. SE estimates, which were obtained by a bootstrap procedure using 1,000 replicates, for the between-group mean number of nucleotide differences are shown above the diagonal. Evolutionary analyses were conducted by using MEGA5.2.2 (http://www.megasoftware.net). Gray shading of mean numbers of nucleotide differences indicates higher percentage identity with darker shaded cells. Influenza A virus isolate names have been shortened to conserve space (OSU, indicates swine isolates; human isolates are identified by their unique number). Fair A, OSU50, OSU52; Fair B, OSU129, OSU138; Fair C, OSU175, OSU176; Fair D, OSU268, OSU293, 17; Fair E, OSU307, OSU420; 57; Fair F, OSU363, OSU370; 38; Fair G, OSU447, OSU450, 56; Fair H, OSU464, OSU467, 62; Fair I, OSU483, OSU484, 71; Fair J, OSU522, OSU527, 80.

**Figure 2:**
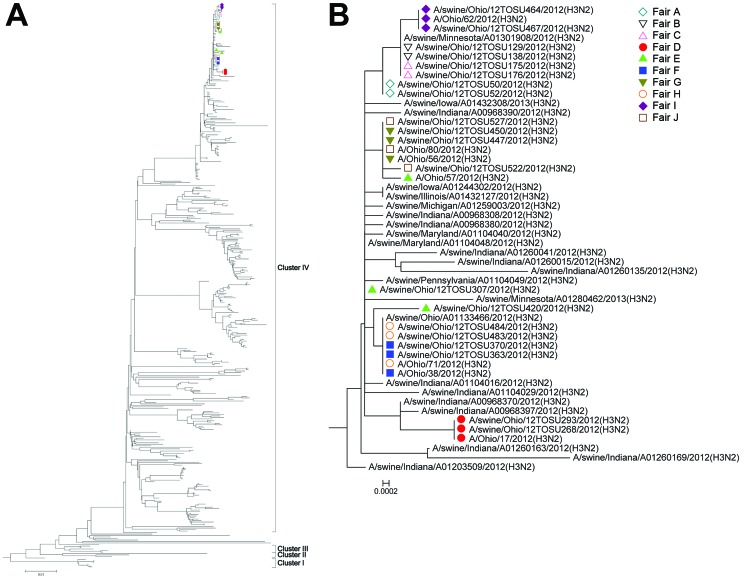
Hemagglutinin phylogeny. A) Phylogenetic relationships of the hemagglutinin sequences of swine-origin subtype H3 influenza A viruses from agricultural fairs, Ohio, USA, 2012. B) Expanded view of isolates. Isolates recovered from swine and humans at the same fair are identified with the same color and symbol. Scale bars indicate nucleotide substitutions per site.

**Figure 3:**
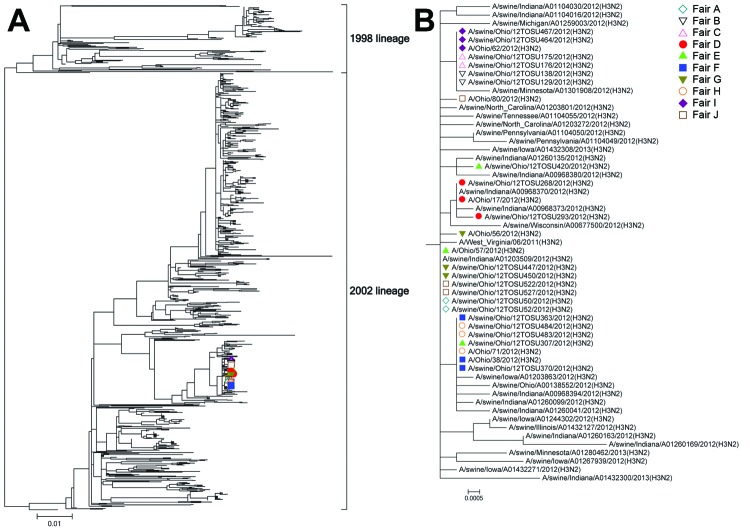
Neuraminidase phylogeny. A) Phylogenetic relationships of the neuraminidase sequences of swine-origin subtype N2 influenza A viruses from agricultural fairs, Ohio, USA, 2012. B) Expanded view of isolates. Isolates recovered from swine and humans at the same fair are identified with the same color and symbol. Scale bars indicate nucleotide substitutions per site.

Phylogenetic analysis of each gene segment of the influenza A(H3N2v) isolates from humans and influenza A(H3N2pM) isolates from swine demonstrated tight clustering with each other (Figures 2, 3, and Technical Appendix). The hemagglutinin segments are typical of cluster IV H3 influenza A viruses circulating among swine in North America (Figure 2, panel A). The neuraminidase segments of the subtype H3N2pM and H3N2v isolates described here clustered tightly together as a sublineage of the 2002 lineage of swine in North America (N2) (Figure 3, panel A). The only other virus included in this neuraminidase sublineage recovered before 2012 was the neuraminidase segment from isolates of subtype H3N2v that caused infection in West Virginia in 2011 (*11*), represented by the A/West Virginia/06/2011(H3N2) isolate (Figure 3, panel B).

## Discussion

The results reported here demonstrate that influenza A(H3N2pM) viruses were commonly circulating among exhibition swine in Ohio during the 2012 agricultural fair season, and genome analysis confirmed zoonotic transmission that resulted in human infection with subtype H3N2v virus. Concurrent infection of swine and humans with swine-origin influenza A(H3N2) virus at Fair D has been reported (*20*), but our molecular investigation demonstrates that the result of that previous study was not an isolated event. Rather, the subtype H3N2pM isolates recovered from swine at 6 additional agricultural fairs in Ohio are molecularly linked to human infections with subtype H3N2v virus contracted at each of those respective fairs. These data provide molecular confirmation of the epidemiologic linkage between cases of subtype H3N2v virus infection and exposure to swine at agricultural fairs.

The fact that all influenza A(H3N2) virus isolates from humans and swine included in this investigation had >99.5% nt identity provides convincing evidence that the same strain of influenza A(H3N2pM) virus was at the 10 Ohio fairs with influenza A virus–positive swine detected in this study. Even within that high degree of similarity among isolates during the 2012 fair season, influenza A(H3N2v) isolates from humans differed from their fair-matched influenza A(H3N2pM) isolates from swine by no more than 16 nt (range 0–16) at 6 of the 7 fairs; the exception was Fair E, for which the closest match between isolates from humans and swine was 34 nt. The 2 sequenced swine-origin subtype H3N2 isolates from Fair E differed from each other by 32 nt, indicating increased genetic diversity among the influenza A viruses circulating among the swine at Fair E compared with that at the other fairs from which isolates from swine differed by ≤7 bases (data not shown). This diversity among isolates from Fair E can be seen in the phylogenetic trees (Figures 2, panel B; Figure 3, panel B; and Technical Appendix). The genetic diversity among the swine-origin influenza A virus isolates from Fair E is probably a result of multiple independent influenza A virus introductions into the swine population; Fair E hosts a larger number of swine (>800), representing a larger geographic area than the other fairs in the study. Additionally, the swine at Fair E were sampled as part of 2 separate load-outs (shipments out of the fair), which resulted in a total of 40 samples from the fair. Because only 2 isolates from swine were sequenced per fair, it is possible that a closer genetic counterpart for the subtype H3N2v isolate from humans exists within the 26 unsequenced isolates from swine at Fair E.

The odds of having cases of influenza A(H3N2v) virus infection in humans were much higher for fairs at which >1 pig was infected with influenza A virus because cases of subtype H3N2v infection in humans were linked only to fairs with swine infected with influenza A virus. Of note is the lack of human H3N2v cases associated with Fairs A, B, and C (Table 1), although the influenza A(H3N2pM) viruses circulating among the swine at those fairs were highly similar to the viruses recovered from swine and humans later in the fair season (Figure 2, panel B). The frequency of virus isolation from swine was ≤45% at Fairs A, B, and C, whereas frequency at the 7 fairs associated with human cases of subtype H3N2v virus infection (Table 1) was >70%. This finding provides a basis for optimism that efforts to decrease the proportion of influenza A virus–infected swine at fairs will decrease the risk to public health. Another possible explanation for the lack of human cases of H3N2v infection during Fairs A, B, and C is that increased awareness and surveillance led to a surveillance artifact caused by previous underdiagnosis and/or underreporting of cases in humans. No human cases of subtype H3N2v infection were reported in Ohio until after the initial reports of such cases in Indiana during July 2012 became public (*26*). After publication of these cases, the Ohio Department of Health began enhanced influenza surveillance, and almost immediately local Ohio public health jurisdictions began alerting the Ohio Department of Health that persons who had been exposed to swine at agricultural fairs were seeking medical care for influenza-like illness.

Influenza A virus–infected exhibition swine threaten public health, and recently, increased emphasis has been placed on educating fair organizers and exhibitors about implementing appropriate precautions when exhibition swine become ill (*27*). Although swine showing clinical signs of influenza-like illness at agricultural fairs are typically removed from public display and/or excused from the exhibition, a previously reported high prevalence of subclinical infection in swine at agricultural fairs (*9*) suggests that many exhibitors and visitors are unknowingly being exposed to swine infected with influenza A virus. In this 2012 investigation, 6 (60%) of 10 agricultural fairs with influenza A–infected swine did not report any influenza-like illness among the exhibition swine (Table 1), further demonstrating the public health risk posed by swine with subclinical influenza infections.

Swine-to-human transmission and human-to-swine transmission of influenza A virus are known to occur at fairs (*28*), highlighting the fact that swine in this setting are potentially exposed to multiple lineages of influenza A viruses simultaneously, making fairs ideal locations for genomic reassortment and novel virus formation. The swine exhibited at agricultural fairs and livestock exhibitions account for a small but distinct subset of the US swine herd, frequently reared in very small herds as part of youth educational programs (*29*) and generally segregated from swine reared for commercial pork production. Influenza A(H3N2pM) viruses are not unique to fairs, and viruses similar to those described in the study reported here have also been detected in commercial swine populations (*30*), indicating that influenza A virus genes and/or whole viruses are shared between commercial and exhibition swine populations. The frequent movement of exhibition swine could serve as a potential pathway for the spread of influenza A virus, which could cause further dissemination of emergent stains in the larger commercial swine population. The rapid dissemination of highly similar subtype H3N2pM viruses among swine at 10 fairs across the state highlights the need to study the transmission dynamics of influenza A viruses within exhibition swine populations. However, although there is certainly fair-to-fair movement of exhibition swine, the role of infected humans spreading these variant viruses fair-to-fair, farm-to-fair, and fair-to-farm needs to be considered and investigated.

The results of this study support previous calls for enhanced surveillance of influenza A viruses among swine, especially at high-risk swine–human interfaces (*31*). In 2012, across the United States, 309 human cases of influenza A(H3N2v) virus infection were reported (http://www.cdc.gov/flu/swineflu/h3n2v-case-count.htm); 306 of these cases occurred during the summer (*19*). Investigations seeking to identify infected swine were frequently hampered by the limitations of retrospectively tracing suspected swine after exhibition. These limitations include not finding and testing the swine until after peak virus shedding and the unavailability of swine for testing after terminal (mandatory harvest) swine exhibitions. The data presented here from active influenza A virus surveillance among exhibition swine, initiated before recognition of cases of subtype H3N2v virus infection, made Ohio uniquely able to investigate the subtype H3N2v virus outbreaks of 2012. Risk factor analyses of fair characteristics and fair-level management practices used during 2012 showed that Ohio fairs with a larger swine inventory were more likely to have had influenza A virus–infected swine during that year (*32*).

Our findings with regard to the swine side of the swine–human interface at fairs seem to be similar to those from previous years in which no human cases of variant influenza virus infection were reported in Ohio. We detected influenza A virus–infected swine at 25% of the fairs tested during 2012, which corresponds with our previous work showing infection at 22.6% of Ohio fairs during 2009–2011 (*9*). The temporal pattern was also similar to that from previous years; fairs with influenza A virus–positive swine were detected sporadically during the early summer, the number peaked in the middle of the season, and none were detected during autumn. Although the seasonal pattern of subtype H3N2v infections in humans in other states was similar to that in Ohio, extrapolation of the findings beyond Ohio will require more robust influenza A virus surveillance in swine at agricultural exhibitions in other states. The sublineage of N2 virus associated with the cases of variant influenza virus infection that occurred during 2012 is clearly within the larger previously described 2002 lineage of N2 virus circulating among swine in North America (Figure 3, panel A) (*30*). The fact that before 2012 this sublineage had only been detected in humans and never in swine further illustrates the need for better influenza A virus surveillance in swine populations.

The sporadic nature in which human infections with variant influenza virus had been reported before 2012 (*10*,*13*,*14*,*33*) highlights an unprecedented frequency of interspecies influenza A virus transmission that occurred during the 2012 outbreak of subtype H3N2v virus infections. Although 306 cases of subtype H3N2v infection were identified during the summer of 2012, thousands more probably went undocumented during the same period (*34*,*35*). Possible explanations for the increased number of variant influenza A virus infections during 2012 include increased transmissibility provided by reassorted gene segments from influenza (H1N1)pdm09 virus (*36*,*37*), limited immunity to swine-origin H3N2 among children <12 years of age (*38*–*40*), and/or increased awareness because of the 2011 cases of subtype H3N2v infection (*15*). During 2013, only 19 cases of infection with subtype H3N2v were reported (http://www.cdc.gov/flu/swineflu/h3n2v-case-count.htm); during that year, public health departments were on the lookout for cases of variant influenza A virus infection. Further evaluation of viral, host, and environmental factors will be needed for elucidation of the difference between the 2012 and 2013 fair seasons.

Whatever the reason for the increased incidence of cases of influenza A(H3N2v) virus infection during 2012, mitigation strategies must be undertaken to decrease the risk for influenza A virus transmission across the swine–human interface at fairs, animal markets, abattoirs, and commercial swine production units. Influenza A virus infections in exhibition swine represent an unquantified public health risk. Rigorous efficacy evaluations and expanded risk assessments of adopted mitigation strategies to protect public and animal health are needed to help animal and public health experts make evidence-based recommendations for reducing intraspecies and interspecies transmission of influenza A virus in this setting. Fair organizers, animal health officials, and public health agencies should take additional steps to decrease the threat to human and animal health. Recently, the National Assembly of State Animal Health Officials and the National Association of State Public Health Veterinarians jointly released some potential measures for fair organizers and exhibitors to consider when hosting and participating in swine exhibitions (*27*). These measures include, but are not limited to, shortening the length of exhibitions, vaccinating swine and humans against influenza A virus infection, promoting awareness at exhibitions, continuous monitoring of swine for signs of influenza-like illness, posting risk-communication signage for visitors to the swine barns, and decreasing movement of swine between fairs. Active communication and partnerships between human and animal health agencies are needed for development and implementation of appropriate prevention and control plans. Local health care providers should be alerted to the possibility that patients with influenza-like illness might have variant influenza A virus infection, especially when agricultural fairs or exhibitions are being held in the community.

Technical Appendix.  Segment sequence identifiers (GenBank or EpiFlu accession nos.) for influenza A viruses and phylogenetic relationships of the sequences.
