# Supplementary material for: Swine-to-Human Transmission of Influenza A(H3N2) Virus at Agricultural Fairs, Ohio, USA, 2012
Source: Emerg Infect Dis. 2014 Sep;20(9):1472–80. doi: 10.3201/eid2009.131082 (PMC4178388; doi:10.3201/eid2009.131082)
Supplement: Supplementary file 1 — Technical Appendix. Segment sequence identifiers (GenBank or EpiFlu accession nos.) for influenza A viruses and phylogenetic relationships of the sequences. [file 13-1082-TechApp-s1.pdf]

Article DOI: http://dx.doi.org/10.3201/eid2009.131082

Swine-to-Human Transmission of Influenza A(H3N2) Virus at Agricultural Fairs, Ohio, USA, 2012

Technical Appendix

Technical Appendix Table

		Segment sequence identifier (GenBank or EpiFlu accession no.)							
Isolate	Fair	PB2	PB1	PA	HA	NP	NA	M	NS
A/swine/Ohio/12TOSU50/2012(H3N2)	A	KC866578	KC866577	KC866580	KC866582	KC866583	KC866579	KC866576	KC866581
A/swine/Ohio/12TOSU52/2012(H3N2)	A	KC866570	KC866569	KC866572	KC866574	KC866575	KC866571	KC866568	KC866573
A/swine/Ohio/12TOSU129/2012(H3N2)	B	KC855513	KC855512	KC855515	KC855517	KC855518	KC855514	KC855511	KC855516
A/swine/Ohio/12TOSU138/2012(H3N2)	B	KC855497	KC855496	KC855499	KC855501	KC855502	KC855498	KC855495	KC855500
A/swine/Ohio/12TOSU175(H3N2)	C	KC855489	KC855488	KC855491	KC855493	KC855494	KC855490	KC855487	KC855492
A/swine/Ohio/12TOSU176(H3N2)	C	KC855521	KC855520	KC855523	KC855525	KC855526	KC855522	KC855519	KC855524
A/swine/Ohio/12TOSU268/2012(H3N2)	D*	JX534958	JX534959	JX534960	JX534961	JX534962	JX534963	JX534964	JX534965
A/swine/Ohio/12TOSU293/2012(H3N2)	D*	JX534966	JX534967	JX534968	JX534969	JX534970	JX534971	JX534972	JX534973
A/Ohio/17/2012(H3N2)	D*	EPI397996	EPI397917	EPI397916	EPI397997	EPI397915	EPI397918	EPI392679	EPI392678
A/swine/Ohio/12TOSU363/2012(H3N2)	F	KC590643	KC590642	KC590645	KC590647	KC590648	KC590644	KC590641	KC590646
A/swine/Ohio/12TOSU370/2012(H3N2)	F	KC590635	KC590634	KC590637	KC590639	KC590640	KC590636	KC590633	KC590638
A/Ohio/38/2012(H3N2)	F	EPI395295	EPI393315	EPI393314	EPI393317	EPI393312	EPI393316	EPI398038	EPI393313
A/swine/Ohio/12TOSU307/2012(H3N2)	E	JX565502	JX565503	JX565504	JX565505	JX565506	JX565507	JX565508	JX565509
A/swine/Ohio/12TOSU420/2012(H3N2)	E	JX565510	JX565511	JX565512	JX565513	JX565514	JX565515	JX565516	JX565517
A/Ohio/57/2012(H3N2)	E	EPI397683	EPI397354	EPI397353	EPI398040	EPI396285	EPI397626	EPI396287	EPI396286
A/swine/Ohio/12TOSU447/2012(H3N2)	G	JX565494	JX565495	JX565496	JX565497	JX565498	JX565499	JX565500	JX565501
A/swine/Ohio/12TOSU450/2012(H3N2)	G	JX565486	JX565487	JX565488	JX565489	JX565490	JX565491	JX565492	JX565493
A/Ohio/56/2012(H3N2)	G	EPI397537	EPI397538	EPI397536	EPI397540	EPI397533	EPI397539	EPI397535	EPI397534
A/swine/Ohio/12TOSU464/2012(H3N2)	I	JX565478	JX565479	JX565480	JX565481	JX565482	JX565483	JX565484	JX565485
A/swine/Ohio/12TOSU467/2012(H3N2)	I	JX565470	JX565471	JX565472	JX565473	JX565474	JX565475	JX565476	JX565477
A/Ohio/62/2012(H3N2)	I	EPI394904	EPI393660	EPI393659	EPI393661	EPI393656	EPI394905	EPI393658	EPI393657
A/swine/Ohio/12TOSU483/2012(H3N2)	H	KC020425	KC020426	KC020427	KC020428	KC020429	KC020430	KC020431	KC020432
A/swine/Ohio/12TOSU484/2012(H3N2)	H	KC020433	KC020434	KC020435	KC020436	KC020437	KC020438	KC020439	KC020440
A/Ohio/71/2012(H3N2)	H	EPI395284	EPI395285	EPI393671	EPI393673	EPI393668	EPI393672	EPI393670	EPI393669
A/swine/Ohio/12TOSU522/2012(H3N2)	J	KC020441	KC020442	KC020443	KC020444	KC020445	KC020446	KC020447	KC020448
A/swine/Ohio/12TOSU527/2012(H3N2)	J	KC020449	KC020450	KC020451	KC020452	KC020453	KC020454	KC020455	KC020456
A/Ohio/80/2012(H3N2)	J	EPI397676	EPI396174	EPI396173	EPI396175	EPI396170	EPI397624	EPI396172	EPI396171

* Fair D was previously described.[18]

MP

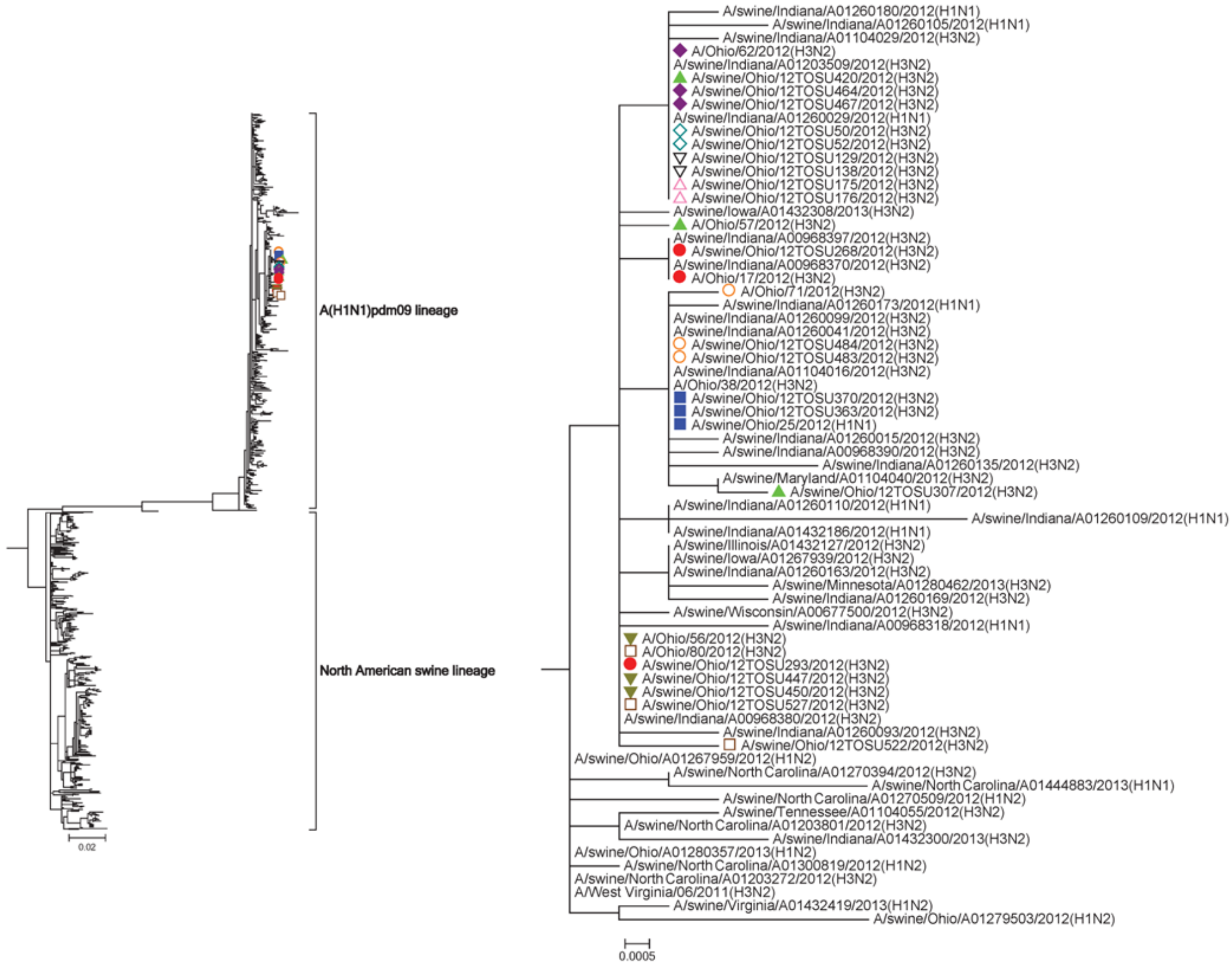

Technical Appendix Figure 1. Left panel: Phylogenetic relationships of the MP sequences. Right panel: Expanded view of isolates from this study, which clustered together with other influenza A viruses containing the MP gene from A(H1N1)pdm09 virus. Isolates recovered from swine and humans at the same fair are identified with the same color and symbol. Fair A, teal open diamond; Fair B, black downward-pointing open triangle; Fair C, pink upward-pointing open triangle; Fair D, red closed circle; Fair E, green upward- pointing closed triangle; Fair F, blue closed square; Fair G, gold downward-pointing closed triangle; Fair H, orange open circle; Fair I, purple closed diamond; Fair J, brown open square.

NP

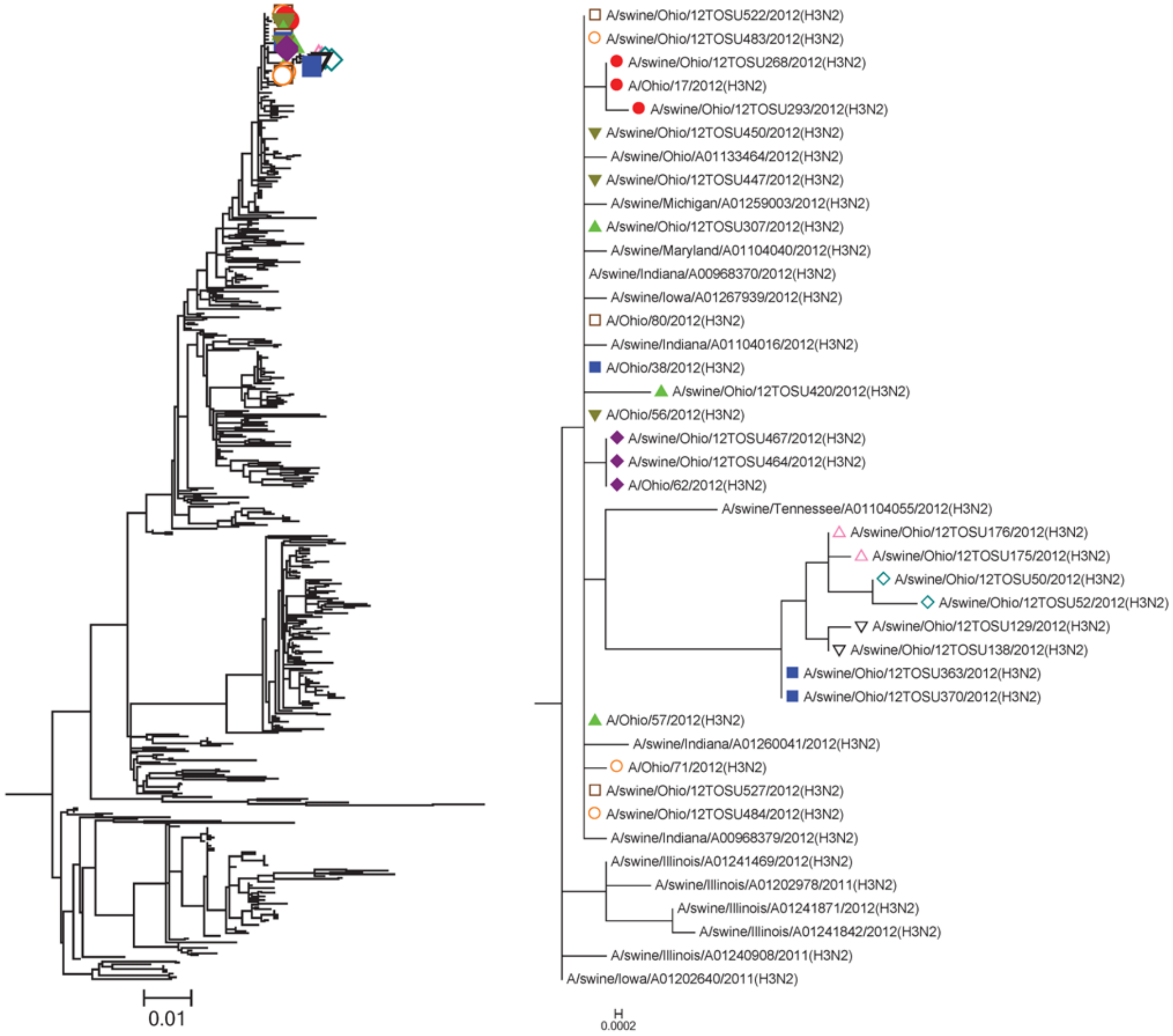

Technical Appendix Figure 2. Left panel: Phylogenetic relationships of the NP sequences. Right panel: Expanded view of isolates from this study. Isolates recovered from swine and humans at the same fair are identified with the same color and symbol. Fair A, teal open diamond; Fair B, black downward-pointing open triangle; Fair C, pink upward-pointing open triangle; Fair D, red closed circle; Fair E, green upward- pointing closed triangle; Fair F, blue closed square; Fair G, gold downward-pointing closed triangle; Fair H, orange open circle; Fair I, purple closed diamond; Fair J, brown open square.

NS

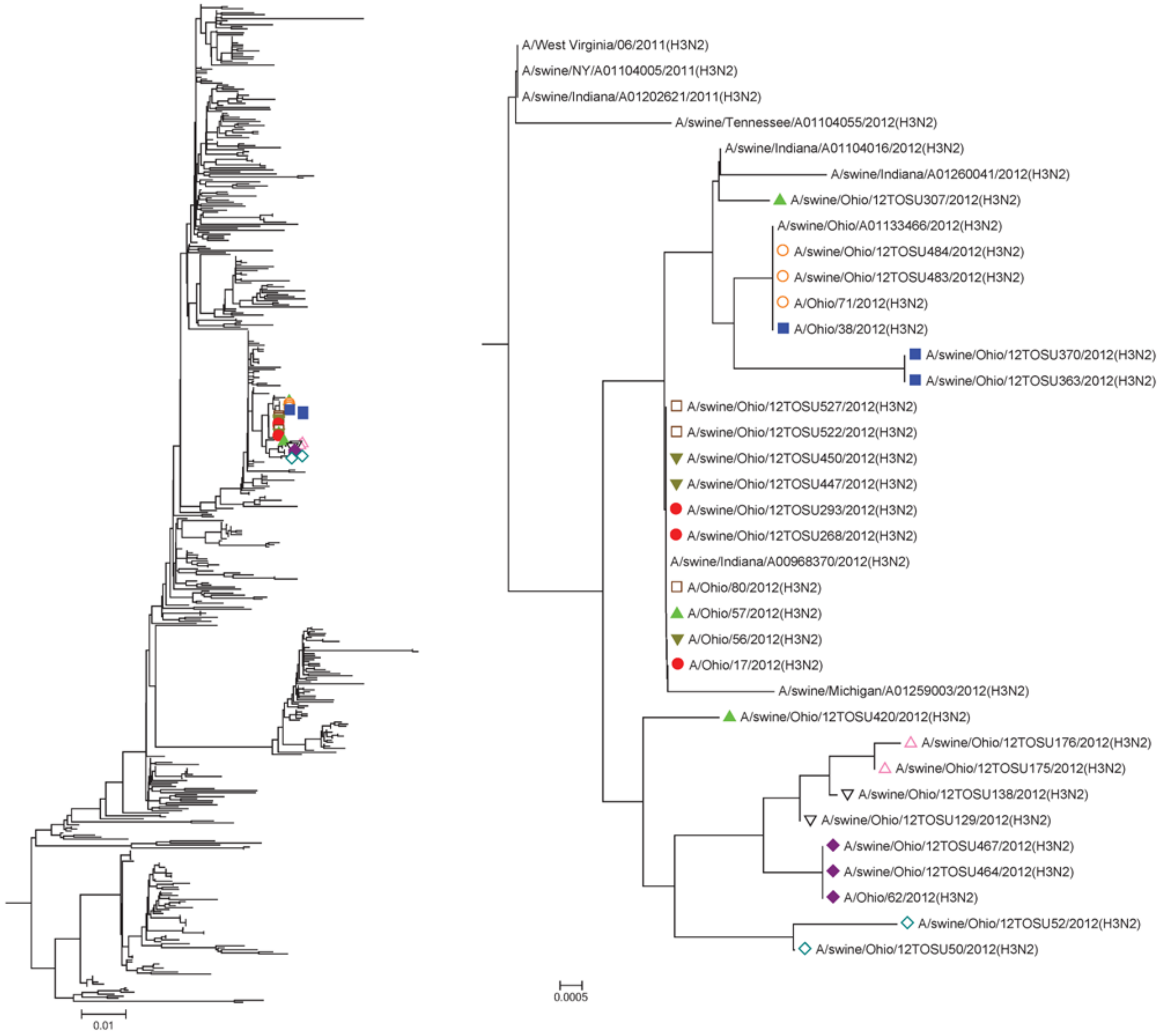

Technical Appendix Figure 3. Left panel: Phylogenetic relationships of the NS sequences. Right panel: Expanded view of isolates from this study. Isolates recovered from swine and humans at the same fair are identified with the same color and symbol. Fair A, teal open diamond; Fair B, black downward-pointing open triangle; Fair C, pink upward-pointing open triangle; Fair D, red closed circle; Fair E, green upward- pointing closed triangle; Fair F, blue closed square; Fair G, gold downward-pointing closed triangle; Fair H, orange open circle; Fair I, purple closed diamond; Fair J, brown open square.

PA

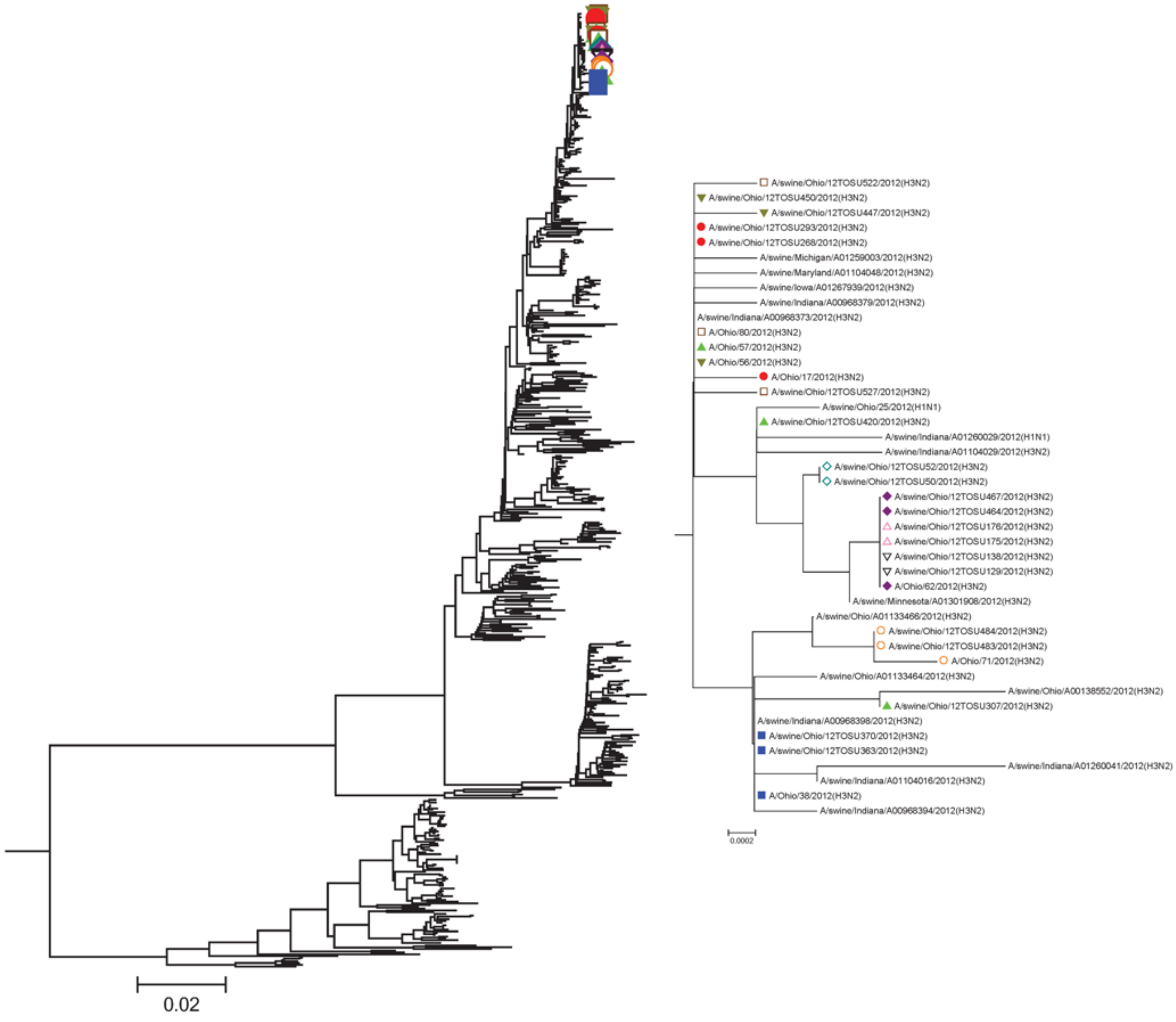

Technical Appendix Figure 4. Left panel: Phylogenetic relationships of the PA sequences. Right panel: Expanded view of isolates from this study. Isolates recovered from swine and humans at the same fair are identified with the same color and symbol. Fair A, teal open diamond; Fair B, black downward-pointing open triangle; Fair C, pink upward-pointing open triangle; Fair D, red closed circle; Fair E, green upward- pointing closed triangle; Fair F, blue closed square; Fair G, gold downward-pointing closed triangle; Fair H, orange open circle; Fair I, purple closed diamond; Fair J, brown open square.

PB1

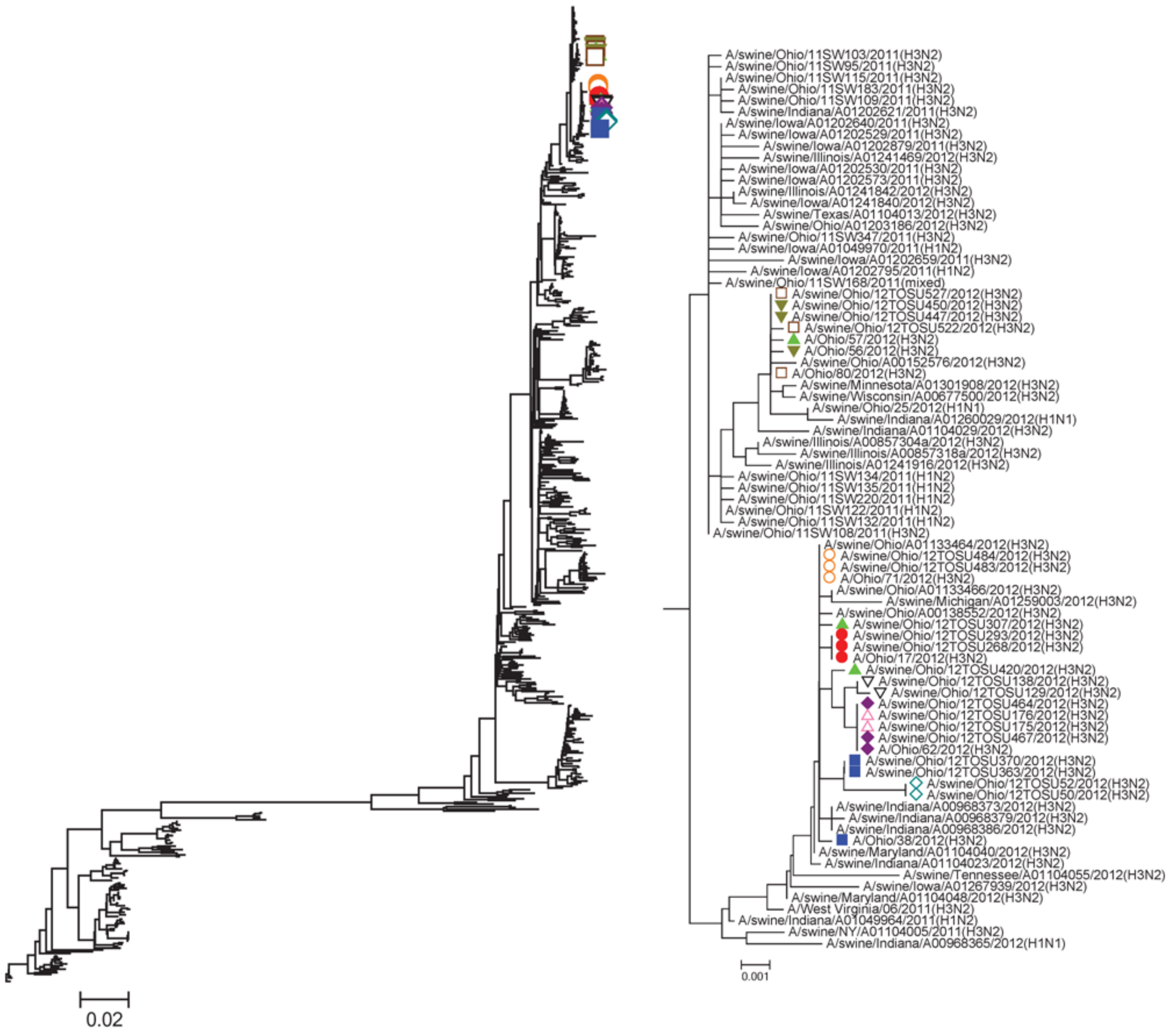

Technical Appendix Figure 5. Left panel: Phylogenetic relationships of the PB1 sequences. Right panel: Expanded view of isolates from this study. Isolates recovered from swine and humans at the same fair are identified with the same color and symbol. Fair A, teal open diamond; Fair B, black downward-pointing open triangle; Fair C, pink upward-pointing open triangle; Fair D, red closed circle; Fair E, green upward- pointing closed triangle; Fair F, blue closed square; Fair G, gold downward-pointing closed triangle; Fair H, orange open circle; Fair I, purple closed diamond; Fair J, brown open square.

PB2

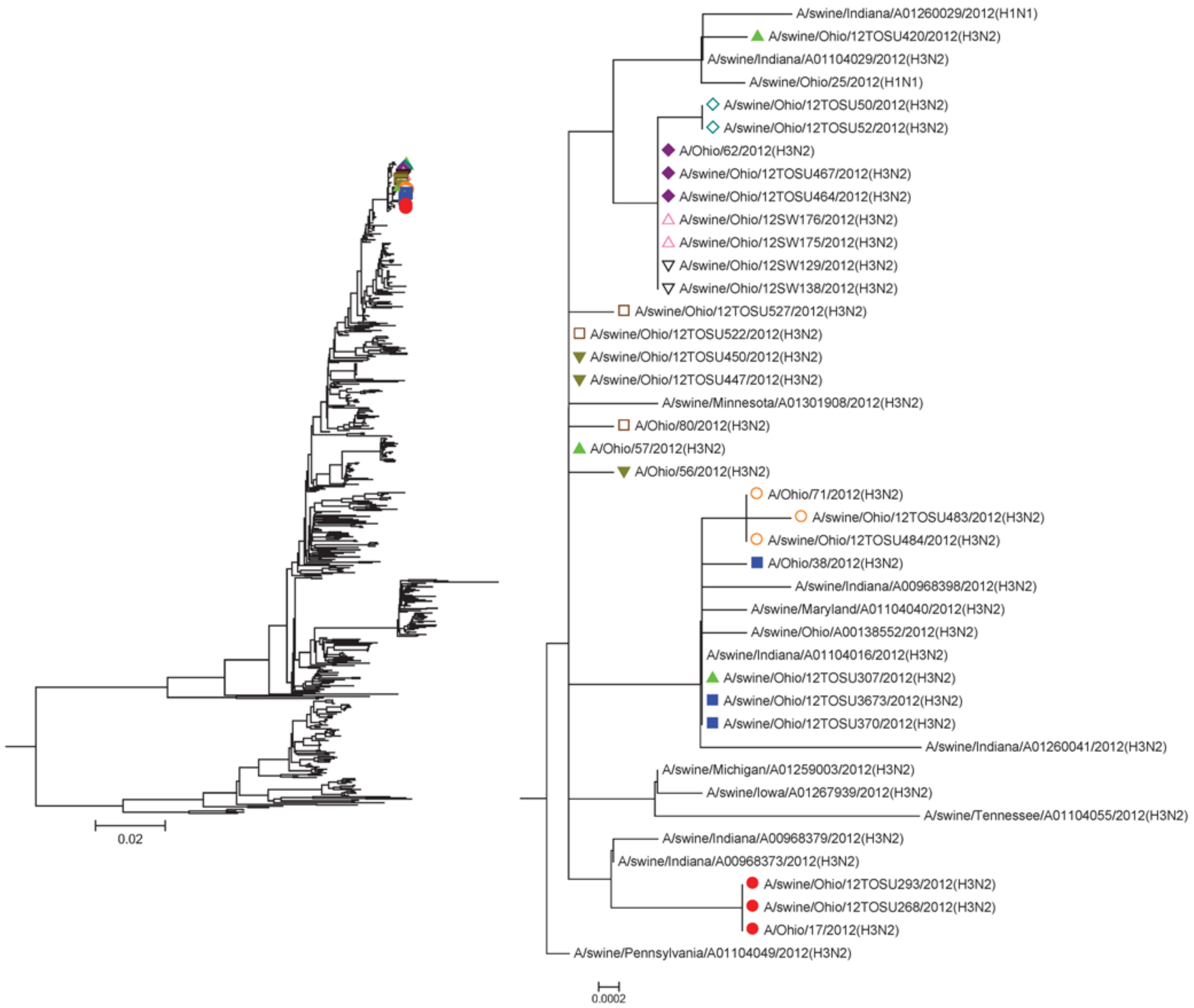

Technical Appendix Figure 6. Left panel: Phylogenetic relationships of the PB2 sequences. Right panel: Expanded view of isolates from this study. Isolates recovered from swine and humans at the same fair are identified with the same color and symbol. Fair A, teal open diamond; Fair B, black downward-pointing open triangle; Fair C, pink upward-pointing open triangle; Fair D, red closed circle; Fair E, green upward- pointing closed triangle; Fair F, blue closed square; Fair G, gold downward-pointing closed triangle; Fair H, orange open circle; Fair I, purple closed diamond; Fair J, brown open square.
